# cDNA Cloning and Molecular Modeling of Procerain B, a Novel Cysteine Endopeptidase Isolated from *Calotropis procera*

**DOI:** 10.1371/journal.pone.0059806

**Published:** 2013-03-20

**Authors:** Abhay Narayan Singh, Prity Yadav, Vikash Kumar Dubey

**Affiliations:** Department of Biotechnology, Indian Institute of Technology Guwahati, Guwahati, Assam, India; University of South Florida College of Medicine, United States of America

## Abstract

Procerain B, a novel cysteine protease (endopeptidase) isolated from *Calotropis procera* belongs to Asclepiadaceae family. Purification of the enzyme, biochemical characterization and potential applications are already published by our group. Here, we report cDNA cloning, complete amino acid sequencing and molecular modeling of procerain B. The derived amino acid sequence showed high sequence homology with other papain like plant cysteine proteases of peptidase C1A superfamily. The three dimensional structure of active procerain B was modeled by homology modeling using X-ray crystal structure of actinidin (PDB ID: 3P5U), a cysteine protease from the fruits of *Actinidia arguta*. The structural aspect of the enzyme is also discussed.

## Introduction

Proteases are the single class of enzymes which occupy a crucial position with respect to physiological role as well as various industrial and therapeutic applications [1]–[3]. Proteases can modify different proteins in highly specific and selective manner for different physiological processes such as activation of zymogenic forms of enzymes by limited proteolysis, blood clotting, lysis of fibrin clots, processing and transport of secretory proteins across the membranes etc [1], [2]. In plants, senescence of leaves, ripening of fruits, seed germination, defense mechanisms against pathogens and several other processes are protease dependent [4]–[6]. Based on the catalytic mechanism, there are seven well-defined classes of peptidases: Serine, cysteine, threonine, metallo, aspartic, glutamic, and asparagine proteases (even though, serine, cysteine, threonine, and metallo peptidases are the four major classes). The first three classes (serine, cysteine and threonine proteases) share a common catalytic mechanism where a particular amino acid (basis of their nomenclature) acts as proton-withdrawing group to promote nucleophilic attack on the peptide bonds. The catalytic mechanism of last two classes (metallo and aspartate protease) is different where an activated water molecule acts as proton-withdrawing group. Occurrence of cysteine proteases is very common in both prokaryotes as well as eukaryotes and in order to properly classify these proteases, total twenty families of cysteine proteases have been identified. Papain like cysteine proteases are modifying enzymes present in wide range of organisms [7]. Initially, the significance of cysteine proteases was limited and they were believed to be involved in some house-keeping activities only. However, owing to its presence in all organisms and involvement at several key steps of physiological and pathological processes to regulate the complex biological networks, the attention on cysteine proteases are magnifying day by day with expanding knowledge and facilities in terms of scientific expertise [6], [8]–[9]. Other than their physiological relevance, proteases are of high commercial value. Proteases are one of the most commercialized enzymes across the globe covering nearly half of the global enzyme sale [10]. Proteases are being utilized in different industries like food industry for preparation of foods for infants, tenderization of meat, clarification of beer, preservation of spices, in several caned juice and soups for enhancement of flavor [11]. In dairy industry, proteases are used for preparation of several dairy products [12]. Likewise in leather and textile industry, proteases are applicable for tannin of leather and smoothening of silk and wool fibers [13]. Proteases with detergents are used for removal of blood and other stains containing proteinaceous ingredients in detergent industry [14]. In pharmaceutical industry, proteases are used with different formulations in several ointments and lotions, as blood coagulant and as food additives [15]–[17].

The plants of Asclepiadaceae family are well known for their latex, containing different components such as alkaloids, terpenoids, tannins, proteins, vitamins and gum etc. It plays an important role in first line of defense from pathogens [4], [5]. *Calotropis procera* of family Asclepiadaceae has been explored for proteases [18]. We have also purified a novel cysteine endopeptidase “Procerain B” from the latex of this medicinal plant and well characterized it in terms of stability and physiochemical properties. Procerain B has high thermal stability and broad pH optima which pronounce its commercial importance [19]. We have also screened some possible applications and found it very applicable in food, dairy, detergent and several other industries [20]. Since procerain B has such a variety of applications, it is desirable to obtain this protein in adequate amount for both basic research and industrial use. The enzyme preparation from plant source depends on several factors such as climatic conditions for the growth of plant and the techniques involved in purification of enzyme, which make traditional purification methods less reliable and efficient for preparation of pure form of enzyme in large quantity. Production of recombinant enzyme is the only option which further offers the possibilities of protein engineering in desired fashion. The recombinant proteins can be engineered in different ways to make it more stable and resistant for autodigestion (common problem in protease research). The specificity of the enzymes can also be altered with selective modifications in recombinant proteins which will further elaborate their applications.

In this study, we aim to find the cDNA sequence of active procerain B for the first time. After confirmation of sequence, the *in silico* analysis was performed and the corresponding cDNA was cloned in two T_7_ promoter based expression vectors (pET-28a(+) and pET-22b(+)) and the three dimensional structure of protein was modeled using a suitable template by homology modeling.

## Materials and Methods

### Materials

The young leaves were collected freshly from the plant grown in IIT Guwahati campus (Guwahati). Trizol, DEPC, isopropanol, DNase, His-Tag Ni affinity beads, IPTG, azocasein and nuclease free water were purchased from Sigma Aldrich (USA). PCR master mix, cDNA synthesis kit, NheI, BamHI, XhoI restriction endonucleases and T_4_ DNA ligase were purchased from New England Biolabs (NEB, England). Plasmid extraction kit, Gel elution kit and PCR cleanup kits were obtained from QUIAGEN (USA). All other chemicals and reagents were of highest purity available.

### Methods

#### RNA isolation and cDNA synthesis

Total RNA was isolated from young leaves of *Calotropis procera* by trizol method. The yield and purity of RNA was determined spectrophotometrically and the quality of RNA was tested on 1% formaldehyde agarose gel. The first strand of cDNA was synthesized by the process of reverse transcription using 1 µg of total RNA as template under following conditions: 40 U M-MuLV reverse transcriptase (NEB, USA) with 0.5 µg of oligo dT_(18)_ primer, 1 mM dNTPs and 20 U RNase inhibitor in a final volume of 20 µl. An aliquot of the first cDNA strand was used as template in PCR reaction for the synthesis of second strand of cDNA and then subsequent amplification of double stranded cDNA was performed with degenerate forward (DEG-N) and oligo dT_(18)_ as reverse primers. The forward degenerate primer was designed on the basis of first seven N-terminal amino acid sequence of procerain B. The PCR was carried out as follows: predenaturation at 95°C, 3 min; 25 cycles of denaturation at 95°C, 30 sec; annealing at 50°C, 45 sec; extension at 72°C, 1 min and a final extension step at 72°C for 10 min.

The amplified products were separated on 1% agarose gel and the product of expected size was extracted from the gel using QIAquick gel extraction kit (QIAGEN, USA). The gel eluted product was ligated to the pTZ57R/T vector using a T/A cloning kit (Fermentas, USA) and the ligation product was transformed into competent *E. coli* DH5α cells. Transformed colonies carrying the TA plasmid with insert were screened by α-complementation of *lacZ* gene and the plasmid DNAs were isolated with QIAprep Spin Miniprep kit (QUIAGEN, USA). The presence of insert DNA in the isolated plasmids were confirmed by PCR with M_13_ primers and finally sequenced from both directions with M_13_ forward and reverse primers.

#### cDNA sequence analysis and construction of expression vector

The cDNA sequence was analyzed to identify its homologous sequences with Basic Local Alignment Search Tool (BLASTx) against non redundant protein sequences in NCBI. The cDNA was translated in all six possible reading frames using ExPASy translation tool (http://web.expasy.org/translate/) to determine the corresponding amino acid sequence. The correct frame was used for BLASTp (http://blast.ncbi.nlm.nih.gov/Blast.cgi) analysis. New specific primers were designed from the sequence and used for subsequent PCR amplification of cDNA. The restriction sites for NheI and BamHI restriction endonucleases (underlined) were introduced at the 5′ of forward (pET28aProB-F) and reverse (pET28aProB-R) primers respectively. The cDNA fragment was amplified by PCR with Dream Taq Green PCR Master Mix (Fermentas, USA) using above mentioned specific primers and checked on 1% agarose gel with EtBr staining. The conditions used for PCR were as follows: predenaturation at 95°C, 3 min; 25 cycles of denaturation at 95°C, 30 sec; annealing at 62°C, 45 sec; extension at 72°C, 1 min and a final extension step at 72°C for 10 min. The PCR product and pET-28a(+) expression vector (Novagen, USA) were digested with NheI (NEB, USA) and BamHI (NEB, USA) restriction endonucleases for the generation of complementary sticky ends and then ligated with T_4_ DNA ligase (NEB, USA). The ligation product was then transformed in competent *E. Coli* DH5α cells by heat shock method and the transformed colonies containing recombinant clones were confirmed by PCR with gene specific primers and double digestion (NheI/BamHI). The recombinant plasmids were isolated from the confirmed colonies and named as pET28_(a)_-ProB. In order to confirm the orientation and presence of the insert in proper frame for the synthesis of complete recombinant protein, the new construct (pET28_(a)_-ProB) was sequenced from both directions with T_7_ promoter and terminator primers.

Simultaneously, the cDNA of procerain B was also cloned in pET-22b(+) expression vector (Novagen, USA) having a pelB leader sequence at N-terminal for periplasmic localization of recombinant protein and His-tag at C-terminal. The cDNA was amplified by PCR with gene specific primers. The restriction sites for BamHI and XhoI restriction endonucleases (underlined) were introduced at the 5′ of forward (pET22bProB-F) and reverse (pET22bProB-R) primers respectively. The recombinant construct (pET22_(b)_-ProB) was confirmed as above and sent for sequencing with T_7_ promoter and terminator primers.

#### Expression and purification of fusion protein

For the expression of fusion protein, *E. Coli* expression strain BL21(DE3) competent cells were transformed with confirmed pET28_(a)_-ProB and pET22_(b)_-ProB recombinant plasmids by heat shock method and the transformed cells from a single colony were grown overnight in Luria Bertani (LB) broth (1% Tryptone, 0.5% Yeast Extract, 1% NaCl), supplemented with 50 µg ml^−1^ kanamycin antibiotic. The overnight grown culture was subcultured in fresh LB medium with 1∶100 dilutions and grown at 37°C with continuous shaking at 180 rpm, until the OD reached 0.6–0.8 at 660 nm. In order to optimize the expression of recombinant procerain B, the transformed culture was induced with 0.1–1.0 mM of isopropyl β-D-thiogalactopyranocyde (IPTG) and incubated at 20, 25 and 37°C. Samples were withdrawn at 2, 4, 6, 8 and 10 h intervals and the expression profile was checked on SDS-PAGE. Un-induced expression of procerain B was also tried. The idea behind un-induced expression (Leaky expression) was to slow down the expression process so that the recombinant protein will have enough time to fold.

After small scale optimization, the expression was scaled up with following conditions. The transformed colonies were grown till mid log phase at 37°C with continuous shaking at 180 rpm, 1.0 mM IPTG was added and then the growth was continued for 6 h at 25°C. The bacterial cells were harvested by centrifugation at 5000 rpm, 4°C for 10 min. The cell pellet was suspended in ice chilled Tris-HCl buffer, pH 7.4 supplemented with sodium tetrathionate (2 mM) and lysozyme (0.2% w/v) and incubated at 4°C for 30 min. The culture was then subjected to sonication with 5 sec on and 9 sec off cycles for 10 min and lysate was centrifuged at 12,000 rpm for 20 min at 4°C. The supernatant (soluble protein) was collected and the pellet (inclusion bodies) was washed twice with wash buffer-1 (20 mM Tris-HCl, 0.5 M NaCl, 10 mM EDTA, 1% Triton X-100) and wash buffer-2 (20 mM Tris-HCl, 0.5 M NaCl, 10 mM EDTA, 0.1% Triton X-100) and then by wash buffer-3 (20 mM Tris-HCl, pH 7.5, 10 mM EDTA). The pellet after washing was dissolved in solubilization buffer pH 7.4 (20 mM Tris-HCl, 0.5 M NaCl, 6 M urea) at 4°C by continuous stirring and then centrifuged at 12,000 rpm for 20 min at 4°C to remove the insoluble traces.

The supernatant of last step was applied on Nickel-affinity column (Ni Sepharose FF, GE HealthCare Life sciences) pre-equilibrated with 10 column volume of solubilization buffer and then washed with five column volume of same buffer with 5 mM imidazole. The bound protein was eluted in same buffer with 250 mM imidazole. As procerain B was expressed with His tag, only procerain B is expected to bind Nickel-affinity column and elute at high imidazole concentration. The eluted protein was tried for refolding with dilution (10 times) by adding the protein drop wise in refolding buffer (20 mM Tris-HCl, pH 8.0, 300 mM arginine, 20 mM cysteine, 5 mM EDTA, 15% (v/v) glycerol) at 4°C and left for overnight with slow stirring and then concentrated by Amicon.

#### Determination of protease activity

The proteolytic activity of recombinant procerain B was determined by the method of Dubey and Jagannadham [19] with slight modifications using azocasein as substrate. The purified recombinant enzyme (20 µg) was incubated at 37°C for 10 min in 500 µl of Tris-HCl buffer pH 7.5 containing 50 mM β-Mercaptoethanol as reducing agent. Azocasein solution (1%, w/v) was prepared in same buffer without β-Mercaptoethanol and added in enzyme solution to make the final volume 1 ml. The solutions were mixed properly and incubated at 37°C for 1 h. TCA (10%, w/v) was added to the reaction mixture to stop the reaction and incubated at room temperature for 5 min. The mixture was centrifuged at 10,000 rpm for 10 min. In case of azocasein as substrate, 500 µl of supernatant was mixed with equal volume of 50 mM NaOH and the color developed was quantified spectrophotometrically at 440 nm. A control assay was performed without enzyme and used as blank in all spectrophotometric experiments.

#### Sequence analysis

The molecular weight, isoelectric point (pI), number of cysteine, tyrosine and tryptophan residues and the number of disulphide bonds in procerain B were calculated with pepstats (http://emboss.bioinformatics.nl/) server and the *in silico* data was compared with biochemical data [19].

#### Homology modeling

The amino acid sequence was used as input for finding the homologous protein sequences with 3D structure using BLASTp (http://blast.ncbi.nlm.nih.gov/Blast.cgi) program against PDB database in NCBI. The amino acid sequence of procerain B was aligned with the identified homologous proteins using Clustal X [21] for understanding the conservation of amino acids throughout the protein family. The three dimensional structure of procerain B (hereafter referred as Pro_B) from the latex of *C. procera* was predicted using actinidin from *Actinidia arguta* (PDB Id: 3P5U) [22] with Modeller 9v7 software (http://www.salilab.org/modeller) using sequence alignment and structural coordinates of the template as input. The initial hundred models generated for procerain B were prioritized on the basis of MOLPDF, discrete optimized protein energy (DOPE) and GA341 score. The final model with reasonable statistics (MOLPDF and DOPE) was validated in SAVES server (http://nihserver.mbi.ucla.edu/SAVES/) and subsequently energy was minimized by Gromacs version package (http://www.gromacs.org/). The final model was analyzed with PROCHECK [23] and ERRAT plot and visually inspected with PyMOL (http://pymol.org/).

## Results and Discussion

### RT-PCR and Cloning in pTZ57R/T Vector

**Figure 1:**
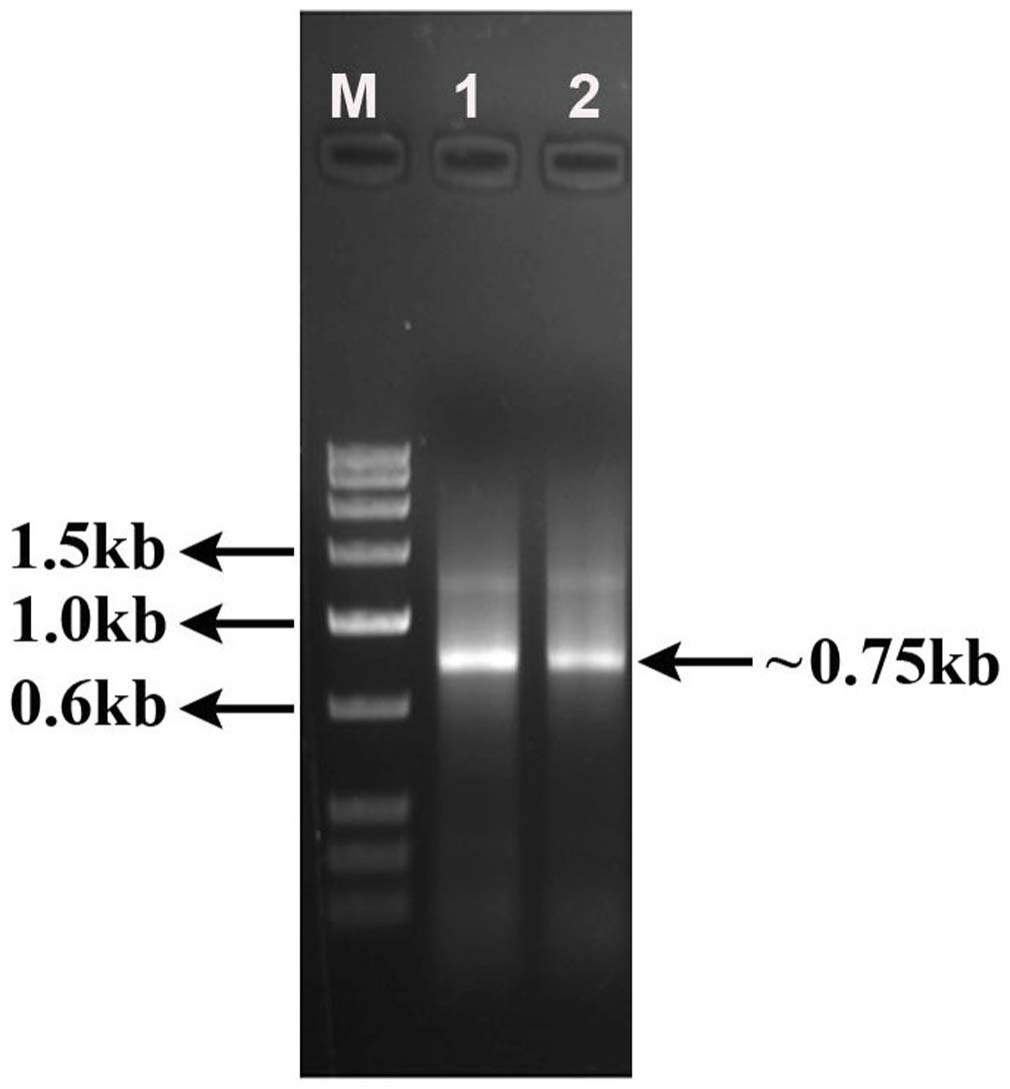
Amplified fragment from a *Calotropis procera* cDNA pool using degenerate forward (based on first seven N-terminal amino acids ofprocerain B) and oligo dT_(18)_ reverse primer. Lane M represents Low range DNA ruler (Bangalore Genei). Lane 1 and 2 represents ∼ 0.75 kb cDNA fragment. One percent agarose gel stained with ethidium bromide.

Total RNA from the young leaves of *C. procera* was extracted by trizole as described in method section and used as template for first strand cDNA synthesis by RT-PCR with oligo dT_(18)_ primer. The subsequent PCR using first strand of cDNA as template with degenerate forward primer and oligo dT_(18)_ as reverse primer **(** **Table 1** **)** yielded an approximately 750 bp fragment **(** **Fig. 1** **)** which was ligated in pTZ57R/T vector to form a pTZ57R/T-ProB recombinant construct.

**Table 1:** Primers used in cloning of procerain B.

S. No.	Name	Sequence (5′-3′)
**1**	DEG-N	CTRCCNAAYTCYGTNGAYTGG
**2**	Oligo dT_(18)_	TTTTTTTTTTTTTTTTTT
**3**	pET28aProB-F	CGGGCTAGCCTGCCTAACTCCGTT
**4**	pET28aProB-R	CCGGATCCTCGAGTCAATAAACAGGATA
**5**	pET22bProB-F	ATGGATCCACTGCCTAACTCCGTTG
**6**	pET22bProB-R	CACCTCGAGATAAACAGGATAAGCAGC

**Figure 2:**
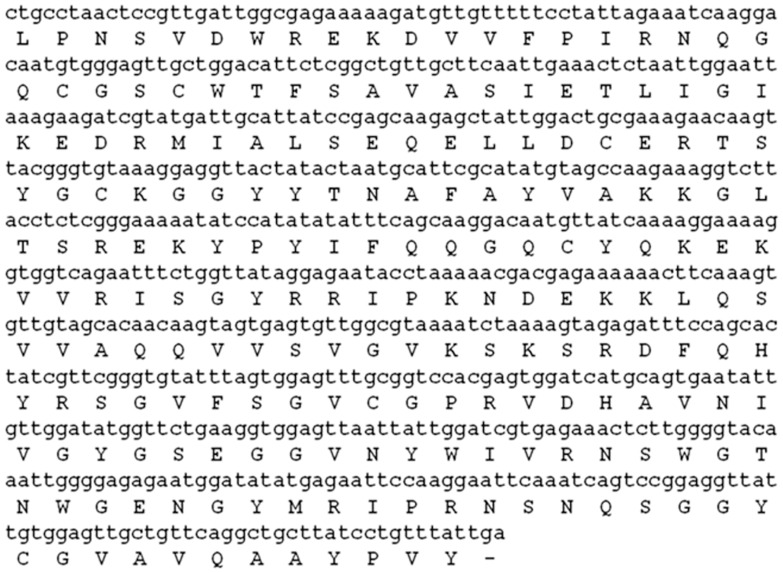
Nucleotide and deduced amino acid sequence of mature Procerain_B from *C. procera*. The amino acid sequence was predicted using TRANSLATE tool of ExPASy server (http://expasy.org/tools/). The (–) denotes the stop codon.

The competent *E. coli* (DH5α) cells were transformed with ligation product and the plasmids isolated from transformed colonies were sequenced. The DNA fragment of 639 bp was identified as a putative cysteine protease by BLASTx analysis as described in earlier section. The resulting cDNA sequence, named as Pro_B was submitted to nucleotide database (Accession number: KC128816). The BLASTx analysis revealed high sequence similarity with pro-asclepain, asclepain cI and asclepain cII isolated from *Gomphocarpus fruticosus* subsp. fruticosus (E value = 2×10^−103^) and *A. curassavica* (E value = 4×10^−101^ and 5×10^−93^). According to the BLAST results the putative protein belongs to peptidase C1A superfamily and the corresponding translated sequence is given in **Fig. 2**. There are couples of mismatches in chemically derived N-terminal sequence and sequence amplified from c-DNA [20]. We have designed degenerate primer using first seven amino acids sequence obtained from Edman’s degradation (chemical method). The c-DNA was amplified using oligo-dT and the degenerate primer. Only two c-DNA were amplified, one 1.25 kb and other 0.75 kb (corresponding to expected mass of procerain B) as shown in Fig. 1. Product of 1.25 kb was also sequenced and found to be tubulin protein while sequence of 0.75 kb matched well with procerain B. The experiment was repeated multiple times. Thus, we concluded that the mismatches at few positions in chemically derived sequence and sequence from c-DNA is likely to be the error of protein sequencing.

### *In silico* Sequence Analysis

The physiochemical properties of procerain B were calculated with pepstats server **(** **Table 2** **)**. The molecular weight of procerain B was predicted to be 23.8 kDa with seven cysteine, fifteen tyrosine and five tryptophan residues in it. Out of seven, six cysteine were found to be involved in the formation of disulphide bond while the active site cysteine (Cys_25_) was free. The isoelectric point of procerain B was identified as 9.25 which reflect the net positive charge on the surface of procerain B at neutral or lower pH. The physiochemical parameters of procerain B slightly differs from biochemical studies reported earlier as the biochemical studies are prone to errors [19]. It is worth mentioning that the enzyme does not have any carbohydrate moiety attached but possibility of other modifications cannot be ruled out [19].

**Table 2:** Physiochemical properties of Procerain B derived from amino acid sequencing data.

S.No.	Properties	Sequence Analysis
**1**	Molecular Weight	23.802 kDa
**2**	Isoelectric Point	9.25
**3**	Number of Cysteine Residues	7
**4**	Number of Disulphide Bonds	3
**5**	Number of Tryptophan Residues	5
**6**	Number of Tyrosine Residues	15

### Sub Cloning in Expression Vectors (pET-28a(+) and pET-22b(+))

**Figure 3:**
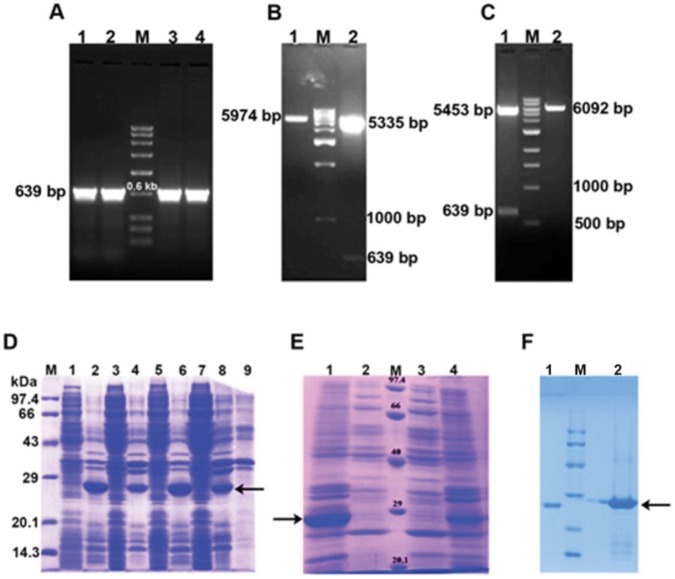
Confirmation of pET28_(a)_ProB and pET22_(b)_ProB clones with PCR and double digestion. (**A**) Lane 1 & 2 represents PCR amplified 639 bp fragment using pET28_(a)_ProB construct as template. M, low range DNA ruler (Bangalore Genei). Lane 3 & 4 represents PCR amplified 639 bp fragment using pET22_(b)_ProB construct as template. (**B**)**.** Lane 1 represents pET28_(a)_ProB construct digested with Nhe1. The DNA fragment of 5974 bp represents the combined size of pET-28a(+) and Procerain B cDNA. Lane M represents 1 kb DNA ladder (NEB). Lane 2 represents release of 639 bp fragment (Procerain B cDNA) on double digestion (Nhe1/BamH1) of pET28_(a)_ProB construct. (**C**)**.** Lane 1 represents release of 639 bp fragment (Procerain B cDNA) on double digestion (BamH1/Xho1) of pET22_(b)_ProB construct. Lane M represents 1 kb DNA ladder (NEB). Lane 2 represents pET22_(b)_ProB construct digested with BamH1. The DNA fragment of 6092 bp represents the combined size of pET-22b(+) and Procerain B cDNA. (**D**) SDS-PAGE showing the over expression of recombinant procerain B in BL21 transformed with pET28_(a)_ProB. Lane M represents medium range protein molecular weight marker (Bangalore Genei). Lane 1 and 2 represent supernatant and pellet of induced culture (1 mM IPTG) at 37°C. Lane 3 and 4 represent supernatant and pellet of un-induced culture at 37°C. Lane 5 & 6 represents supernatant and pellet of induced (1 mM IPTG) at 25°C. Lane 7 and 8 represent supernatant and pellet of uninduced culture at 25°C. Lane 9 represents pellet of BL21 transformed with pET28a(+) at 25°C. (**E**)**.** Expression profile of recombinant procerain B in BL21 transformed with pET22_(b)_ProB. Lane 1 and 2 represent pellet and supernatant of induced culture (1 mM IPTG) at 25°C. **M**. Medium range protein molecular weight marker (Bangalore Genei). Lane 3 and 4 represent supernatant and pellet of un-induced culture at 20°C. (**F**)**.** Comparison of native and recombinant procerain B (with His-tag) on SDS-PAGE. Lane 1 represents purified native procerain B. Lane M represents medium range protein molecular weight marker (Bangalore Genei). Lane 2 represents purified recombinant procerain B (with His-tag). Slight difference in molecular weight of native and recombinant procerain B is due to presence of His-tag in recombinant procerain B. Arrow in Figure 3 (D, E, F) indicates recombinant procerain B.

In order to express a recombinant protease, the cDNA fragment was amplified with gene specific forward and reverse primers containing appropriate restriction sites at their 5′ ends and sub-cloned in pET-28a(+) and pET-22b(+) expression vectors as described in earlier section. The plasmids were isolated from the recombinant colonies and the presence of cDNA fragment in recombinant pET28_(a)_-ProB and pET22_(b)_-ProB vectors was confirmed by PCR and double digestion **(** **Fig. 3** **)**. The amplification of 639 bp fragments in PCR **(** **Fig. 3A** **)** and release of same size of fragment by restriction digestion **(** **Fig. 3B** **and** **Fig. 3C** **)** confirms the presence of cDNA fragment in recombinant pET28_(a)_-ProB and pET22_(b)_-ProB vectors. The presence of same cDNA fragment in correct frame for complete expression of recombinant protein was confirmed by sequencing with T_7_ forward and T_7_ reverse primers which resulted in frame sequence with proper 5′-3′ orientation. The *in silico* translated cDNA sequence predicted an open reading frame of 212 amino acids.

### Expression and Purification

The recombinant endopeptidase (procerain B) was expressed in BL21 (DE3) strain of *E. coli* at optimized induction, 1.0 mM IPTG at 25°C for 6 h, and then the culture was centrifuged and sonicated to release the proteins. The SDS-PAGE analysis of lysate reflected the newly synthesized recombinant protein as major fraction of total lysate protein. For soluble expression of recombinant protein, the endopeptidase was expressed with **(** **Fig. 3E** **)** and without **(** **Fig. 3D** **)** pelB leader sequence. The pelB sequence at N-terminal was supposed for translocation of recombinant protein to periplasm where the microenvironment will be less reducing in comparison to cytoplasm for disulphide bond formation which will favor the proper folding of protein. Most of the recombinant endopeptidase was expressed in form of inclusion bodies present in pellet. Different other modified strains (*Rosetta-gami, Rosetta 2*-(DE3)) were also tried for soluble expression of recombinant endopeptidase but the protein was expressed in form of inclusion bodies (data not shown). The inclusion bodies were dissolved with 6 M urea and purified by Ni-affinity chromatography. The purified protein was tried to refold by dilution as described in earlier section to fold the protein in proper orientation for proteolytic activity. The approximate yield of recombinant procerain B was nearly 2.5 mg L^−1^.

In order to determine the biological activity of recombinant procerain B, the proteolytic assay was performed with both folded and unfolded protein as described in method section. Both forms of recombinant endopeptidase did not show any activity, while the native form of procerain B was proteolytically active. The detailed study of native procerain B was reported earlier by our group [19]. Finally, procerain B was solubilised from inclusion bodies using different concentrations of denaturants; urea and guanidine hydrochloride. It was found that 6 M urea was most effective in solubilising procerain B from inclusion body. Attempts to oxidative refolding of enzyme were also made using reduced and oxidised glutathione, but enzyme was still found in non-active form. The lack of proteolytic activity may be attributed to misfolding of protein due to the absence of proper folding conditions in host system. Literature survey suggests the importance of pro-peptide region in proper folding of several cysteine proteases. The absence of pro-peptide region could be another possibility for the lack of proteolytic activity in recombinant enzyme. Another reason which could affect the proper folding of protein is the presence of non cysteine protease region (from vector) in the recombinant protein. Although these regions are very small in size but may prevent the proper folding of protein. The other possibility is the requirement of a unique folding condition different from the other known cysteine proteases. Therefore the reason for absence of biological activity in recombinant endopeptidase is not clear and further research is required in this direction.

### Homology Modeling

**Figure 4:**
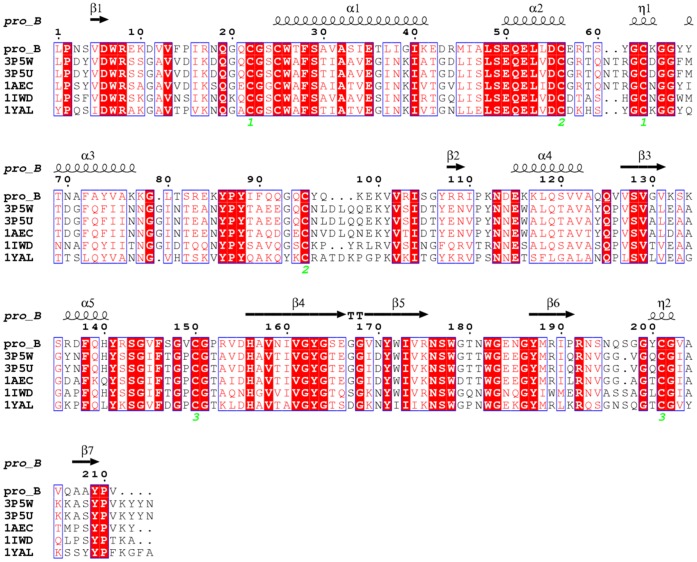
Multiple sequence alignment of translated Pro_B sequence with five top hits of BLASTp analysis (PDB ID: 3P5W, 3P5U, 1AEC, 1IDW and 1YAL) by CLUSTAL V. The active site residues (Cys_25_, His_156_, Asn_176_) and other crucial conserved amino acids are highlighted in red. Three disulphide binding sites are marked in green as 1–1, 2–2, 3–3. The regions of α-helix, β-sheets and η-turns are marked on the top.

**Figure 5:**
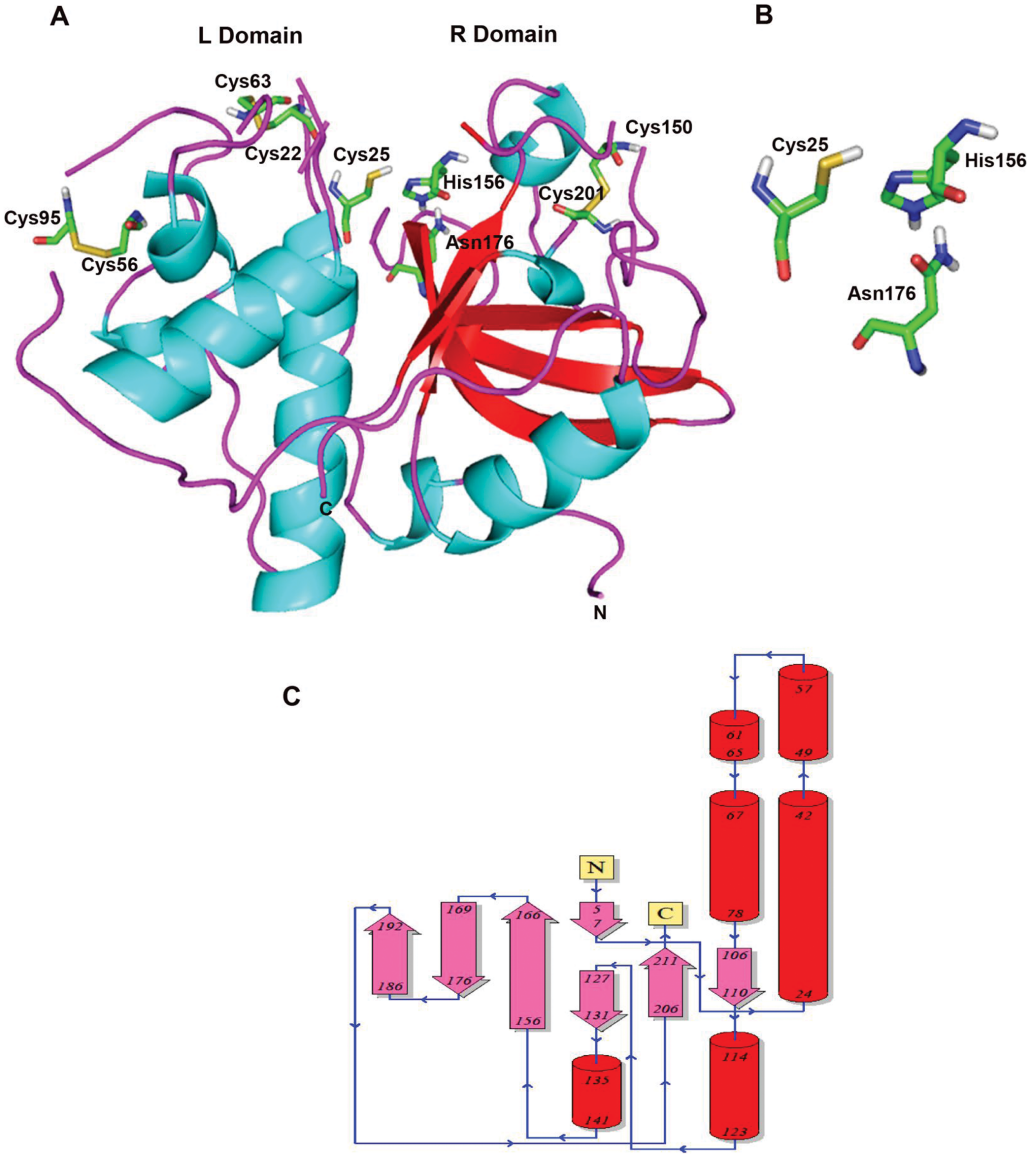
The three dimensional structure of Procerain B. (**A**) Modeled structure with Modeller software using the X-ray crystal structure of actinidin (PDB ID: 3P5U) from the fruits of *Actinidia arguta* as template for homology modeling. The structural analysis of the model revealed that the Pro_B belong to α+β class of proteins with characteristic cysteine proteinase fold consists of one α-helix and four strands of anti-parallel β-sheet which holds the catalytic triad (Cys_25_, His_156_, Asn_176_). The model contains two typical L (N-terminal) and R (C-terminal) domains of papain like cysteine proteases sharing “V” shaped active site. (**B**) The active site residues (Cys_25_, His_156_, Asn_176_) of procerain B. (**C**) Predicted secondary structure of procerain B using PDBSUB server showing two domains of protein. The N-terminal domain is dominated by α-helixes (red) while the C-terminal domain is dominated by anti-parallel β-sheets (pink).

Sequence analysis of Pro_B showed maximum homology with plant cysteine proteases of peptidase C1A superfamily **(** **Fig. 4** **)**, which are highly similar to papain with endopeptidase activity and have preference towards substrates having bulky hydrophobic or aromatic residues at P2 position. Pro_B showed 68% similarity (52% identity) with actinidin, a cysteine protease from the fruits of *Actinidia arguta* belonging to Asclapidaceae family which has multiple crystallized structures (PDB ID: 3P5U, 3P5V, 3P5W, 3P5X) [22]. Owing to its high sequence similarity with query coverage of 99%, conservation of catalytic domains and resolution of crystal structure (1.5 Å), actinidin from *Actinidia arguta* (PDB ID: 3P5U, A chain) was selected as template to model the three dimensional structure of procerain B. The complete structure of Pro_B was modeled except the C-terminal tryptophan which was believed not to participate in structure or function. The quality of final model was validated by Ramachandran plot using PROCHECK and ERRAT plot. PROCHECK analysis revealed that 84.9% residues were falling in most favored region, 14% in additionally allowed regions and only 0.6% (2 residues) was found in generously allowed and disallowed regions. The overall quality factor of the model from ERRAT plot was 90.15 which reflect that the modeled structure has low steric hindrance and is well in the range of good statistics (most of the residues were below 95% cutoff of error-value). The 3D model of Pro_B was energy minimized to eliminate any artifacts in the modeled structure and shown in **Fig. 5A**. The closer structural analysis revealed that the Pro_B belongs to α+β class of protein with characteristic cysteine proteinase fold consisting of one α-helix and four strands of anti-parallel β-sheet which holds the catalytic triad (Cys_25_, His_156_, Asn_176_). Structural comparison of the modeled structure with template showed that both the proteins were superimposed completely with RMSD (Root mean square deviation) of 0.54 Å (Cα atom of 180) and 0.81 Å (Cα atom of 199). The model contains two typical L and R domains of papain like cysteine proteases sharing “V” shaped active site **(** **Fig. 5B** **)**. The topology of the modeled structure was determined by the secondary structure model from PDBsum (http://www.ebi.ac.uk/pdbsum/) **(** **Fig. 5C** **).** The L domain (N-terminal) is populated by three α helices (Ser_24_-Glu_42_, Ser_49_-Arg_57_, Tyr_67_-Lys_78_) while the R domain (C-terminal) mainly composed of a β-motif with anti-parallel β-strands. The active site cysteine (Cys_25_) of Pro_B was present at the N-terminal of longest α helix (17 amino acids) which has more freedom to form ion pair with His156 located in the β-barrel domain of other side. High thermal stability of procerain B (up to 70°C) may be attributed by more number of γ-turns (Gly_20_-Cys_22_, Glu_42_-Arg_44_, Gly_144_-Phe_146_, Pro_152_-Val_154_, Gly_183_-Asn_185_) as compared to actinidin (Gly_20_-Cys_22_, Cys_22_-Ser_24_, Thr_158_-Val_160_, Gly_189_-Glu_191_). The increased thermostability of proteins with γ-turns may be explained by intramolecular hydrogen bonding in short γ-turns [24]. The L domain was supported by two disulphide bonds (Cys_22_-Cys_63_, Cys_56_-Cys_95_) where as the third disulphide bond (Cys_150_-Cys_201_) was present in R domain is conserved as it was observed in other cysteine proteases. The active site analysis around catalytic Cys_25_ with 4 Å radius revealed Thr_27_ as S_2_ position, responsible for substrate specificity. The substrate binding pocket of Pro_B was found to be less hydrophobic (Thr_27_) as compared to actinidin (Ala_27_) and several other proteases of this family.

### Conclusion

Cloning and expression of a cysteine endopeptidase from *Calotropis procera* is reported for the first time. The recombinant protein was expressed as a fusion protein with N-terminal His-tag and with N-terminal pelB sequence for periplasmic translocation. The protein was expressed in form of inclusion bodies. However, we have been able to derive complete amino acid sequence. Further, the three dimensional structure of protein was modeled using actinidin X-ray crystal structure (3P5U) as template and analyzed for conservation of domains with catalytic residues specific for papain like cysteine proteases.
